# The Effects of Terminal Tagging on Homomeric Interactions of the Sigma 1 Receptor

**DOI:** 10.3389/fnins.2019.01356

**Published:** 2019-12-19

**Authors:** Hideaki Yano, Leanne Liu, Sett Naing, Lei Shi

**Affiliations:** Computational Chemistry and Molecular Biophysics Unit, National Institute on Drug Abuse – Intramural Research Program, National Institutes of Health, Baltimore, MD, United States

**Keywords:** sigma 1 receptor, western blot, oligomerization, bioluminescence resonance energy transfer, SDS-PAGE

## Abstract

The sigma 1 receptor (σ1R) has been implicated in cancers, neurological disorders, and substance use disorders. Yet, its molecular and cellular functions have not been well-understood. Recent crystal structures of σ1R reveal a single N-terminal transmembrane segment and C-terminal ligand-binding domain, and a trimeric organization. Nevertheless, outstanding issues surrounding the functional or pharmacological relevance of σ1R oligomerization remain, such as the minimal protomeric unit and the differentially altered oligomerization states by different classes of ligands. Western blot (WB) assays have been widely used to investigate protein oligomerizations. However, the unique topology of σ1R renders several intertwined challenges in WB. Here we describe a WB protocol without temperature denaturization to study the ligand binding effects on the oligomerization state of σ1R. Using this approach, we observed unexpected ladder-like incremental migration pattern of σ1R, demonstrating preserved homomeric interactions in the detergent environment. We compared the migration patterns of intact σ1R construct and the C-terminally tagged σ1R constructs, and found similar trends in response to drug treatments. In contrast, N-terminally tagged σ1R constructs show opposite trends to that of the intact construct, suggesting distorted elicitation of the ligand binding effects on oligomerization. Together, our findings indicate that the N-terminus plays an important role in eliciting the impacts of bound ligands, whereas the C-terminus is amenable for modifications for biochemical studies.

## Introduction

The sigma 1 receptor (σ1R) is a structurally unique transmembrane protein found in the endoplasmic reticulum (ER) and is associated with intracellular membranes in which it has been posited to act as a molecular chaperone (Su et al., 2016). Although it is broadly distributed in many different tissues and cell types, it is particularly enriched in the central nervous system (Weissman et al., 1988). σ1R has been implicated in cancer, neurodegenerative diseases, and psychostimulant abuse, and is thus considered a promising therapeutic target (Maurice and Su, 2009). Despite its physiological and pharmacological significance, the structure-function relationships of σ1R are poorly understood. The recent crystal structures of σ1R in complexes with a variety of ligands provide the foundation for mechanistic elucidation of its function at the molecular level (Schmidt et al., 2016, 2018). They shed light on the conformation and homomeric status of σ1R in the membrane environment. However, functional categorizations of σ1R ligands remain challenging (Katz et al., 2017). Both the differences in intra- or extracellular environments in various σ1R assays and the lack of functional readouts detecting changes in σ1R itself may have contributed to the difficulty in reaching consensus conclusions. Thus, it begs the need for an assay that directly monitors changes occurring in σ1R.

By focusing on σ1R itself, we have developed a bioluminescence resonance energy transfer (BRET)-based assay to categorize σ1R ligands based on presumed changes in σ1R-σ1R interactions; for instance, haloperidol increases the σ1R-σ1R interaction while (+)-pentazocine decreases it (Yano et al., 2018). However, the underlying molecular mechanism could not be directly derived from the BRET signal changes. Instead, the specific oligomerization states were characterized by western blot (WB) (Yano et al., 2018). This biochemical approach has the advantages of being capable of quantifying the sizes and intensities of migration bands to provide stoichiometric information on homomeric oligomerization, and of allowing comparison across tightly controlled experimental conditions or different pharmacological treatments. In both BRET and WB, σ1R has been subjected to end tagging. However, studies have shown that a bulky protein tag on the N-terminus yields aberrant localization and function, while the C-terminal protein tagging appeared to be permissive and largely retained its function (Hayashi and Su, 2003).

In the current study, we investigated the effects of N- and C-terminal tagging on the cellular function of σ1R, in particular its homomeric interactions, in order to evaluate the validity of using such constructs *in vitro* assays. We optimized a WB protocol to detect ligand-induced σ1R oligomerization and compare the results for terminally tagged and unmodified wildtype (WT) σ1R constructs.

## Materials and Methods

### DNA Constructs, Transfection, and Cell Culture

HEK293T Δσ1R cells were generated using the CRISPR-Cas9 gene deletion method (Santa Cruz). Human σ1R is tagged in pcDNA3.1 plasmid with Myc, NanoLuciferase (Nluc), or mVenus, either N-terminally or C-terminally in frame without any linker (Myc-σ1R, Nluc-σ1R, σ1R-Myc, σ1R-Nluc, or σ1R-mVenus). All constructs were confirmed by sequence analysis. For western blot and radioligand binding, 5 μg (otherwise noted) of terminally tagged and unmodified σ1R plasmid was transfected using lipofectamine 2000 (Invitrogen) for HEK 293T Δσ1R cells in a 10 cm plate. For drug induced BRET, a constant amount of total plasmid cDNA (15 μg) in 1:24 (donor:acceptor ratio for σ1R-Nluc and σ1R-mVenus) was transfected in HEK 293T Δσ1R cells using polyethylenimine (PEI) in a 10 cm plate. Cells were maintained in culture with Dulbecco’s modified Eagle’s medium supplemented with 10% fetal bovine serum and kept in an incubator at 37°C and 5% CO_2_. Experiments were performed approximately 48 h post-transfection.

### Western Blot

HEK293T Δσ1R cells were grown as reported (Yano et al., 2018) and transiently transfected with the unmodified σ1R, N-terminally tagged Myc-σ1R, N-terminally tagged Nluc-σ1R, C-terminally tagged σ1R-Myc, or C-terminally tagged σ1R-Nluc in 10 cm plates. After 48 h of growth, confluent cells were harvested in Hank’s Balanced Salt Solution (HBSS), centrifuged at 900 × *g* for 8 min, and resuspended in HBSS. The cells were then incubated in 1 μM haloperidol, 1 μM PD 144418, 10 μM (+)-pentazocine, or 1% DMSO vehicle for 1 h at room temperature. The samples were then centrifuged at 900 × *g* for 4 min and resuspended in lysis buffer [150 mM NaCl, 1.0% triton X-100, 0.5% sodium deoxycholate, Tris 50 mM, pH 7.5, and protease inhibitors (Roche, catalog# 11697498001)]. In the case of mouse tissue preparation, a cortex was dissected out, washed in phosphate buffered saline (PBS), and homogenized in lysis buffer with tissue homogenizer. The samples were sonicated, incubated on ice for 30 min, and centrifuged at 20,000 × *g* for 30 min. Supernatants were transferred to new tubes. Protein concentrations of the supernatants were determined with Bradford protein assay (Bio-Rad, Hercules, CA, United States). Supernatants were mixed with 4×β-mercaptoethanol Laemmli sample buffer to a final 25 μg protein/sample. Samples were electrophoresed at 100 V for 10 min (stacking gel) and 150 V for 30 min (resolving gel) on 10% polyacrylamide Tris-glycine gels (Invitrogen) with running buffer (25 mM Tris, 192 mM glycine and 0.1% SDS, pH 8.3, Invitrogen). Proteins were transferred to PVDF membranes (Invitrogen, catalog# IB24002) for 10 min at 20 V using dry transfer apparatus (Invitrogen, catalog# IB21001) and immunoblotted with antibodies in tris-buffered saline with 0.1% Tween 20. Anti-GAPDH or anti-actin was used as a loading control. The product information and dilutions of primary and secondary antibodies used are summarized in Supplementary Table S1. Blots were imaged using Odyssey LI-COR scanner and analyzed with LI-COR Image Studio^TM^. Photon counts were tabulated for each band density and normalized to the GAPDH band of the same lane. Further, those values are normalized to the summation of all band densities within the same lane as one in percent population graphs in segmented red and blue bars. Fold change bar graphs for different ligands were prepared with the photon counts (not the segmented percentage values) normalized against DMSO vehicle as mean ± SEM.

### Radioligand Binding Assay

Membrane fraction of HEK293T Δσ1R cells was prepared as previously described (Yano et al., 2018). The radioligand incubation was carried out in 96-well plates containing 60 μL fresh Earle’s Balanced Salts Solution (EBSS) binding buffer (8.7 g/l Earle’s Balanced Salts without phenol red (United States Biological) and 2.2 g/L sodium bicarbonate, pH to 7.4), 20 μL of (+)-pentazocine (varying concentrations), 100 μL membranes (25 μg/well total protein), and 20 μL of radioligand diluted in binding buffer (1 nM [^3^H]-(+)-pentazocine (American Radiolabeled Chemicals), final concentration) for each well. We used 1 mM (+)-pentazocine as a non-specific control. Concentrations for non-radioactive (+)-pentazocine in homologous competition assay were: 100 μM, 10 μM, 1 μM, 100 nM, 10 nM, 1 nM, and 0.1 nM in EBSS with 10% DMSO. Total binding was determined with 1% DMSO vehicle (final concentration). All compound dilutions were tested in triplicate, and samples were incubated for 120 min at room temperature. The reactions were terminated by filtration through PerkinElmer Uni Filter-96 GF/B, presoaked in 0.05% PEI for 120 min, and the 96-well filter plates were counted in PerkinElmer MicroBeta Microplate Counter as described (Yano et al., 2018) with counter efficiency at 31% for [^3^H]-(+)-pentazocine. K_d_ and B_max_ values were determined from at least three independent experiments.

### Bioluminescence Resonance Energy Transfer (BRET) Assay

Drug-induced BRET is conducted as optimized previously (i.e., transfection and experimental conditions) (Urizar et al., 2011; Yano et al., 2018). Briefly, cells were prepared in 96-well plates. 5 μM coelenterazine h was added to each well. Three minutes after addition of coelenterazine h (Nanolight), ligands [(+)-pentazocine (Sigma), PD 144418 (Tocris), and haloperidol (Tocris)] were added to each well in serial dilution. BRET was measured as a ratio between measurements at 535 nm for fluorescence (gain 2500, interval time 0.9) and at 485 nm for luminescence (gain 2500, interval time 0.9) using a PHERastar FSX reader (BMG). Results are calculated for the BRET change (BRET ratio for the corresponding drug minus BRET ratio in the absence of the drug). E_max_ values are expressed as the basal subtracted BRET change in the dose-response graphs.

### Animals

Wildtype (WT) C57BL/6J mice were used for σ1R detection in western blot experiments. Animals were housed with food and water available *ad libitum* in temperature (22 ± 2°C)- and humidity (45 ± 5%)-controlled rooms and were maintained on a 12 h light/dark cycle (lights on: 7:00 a.m.–7:00 p.m.). They were experimentally naive at the start of the study and were maintained in accordance with NIH guidelines. All studies were approved by the Institutional Care and Use Committee of the Intramural Research Program, National Institute on Drug Abuse.

### Statistical Analysis

Statistical analyses were performed using GraphPad Prism version 7.0 (GraphPad Software, San Diego, CA, United States). For Figures 2C,F,I, 4C,F, the statistical comparison was obtained by one-way ANOVA followed by *post hoc* Tukey analysis (^∗^*p* < 0.05, ^∗∗^*p* < 0.01, ^∗∗∗^*p* < 0.005, and ^****^p < 0.001).

## Results

### The Unmodified WT Shows Distinct Bands up to Densities Corresponding to Octamers

In this study, we used an antibody-based western blot (WB) approach to detect the configurational changes of σ1R oligomerization in response to ligand binding and to the modification of the N- or C-terminus. To exclude the confounding effects of endogenous σ1R, we heterologously expressed (i.e., rescued) various σ1R constructs in σ1R-null (Δσ1R) HEK293T cells (see section “Materials and Methods”).

**FIGURE 1:**
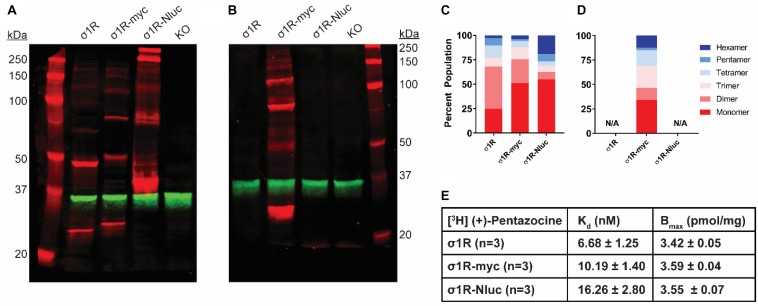
SDS-PAGE mobility comparison among the unmodified and C-terminally tagged σ1R constructs. **(A,B)** Red bands are visualized by anti-σ1R **(A)** or anti-myc **(B)** antibodies on 10% SDS-PAGE (protein standard, σ1R, σ1R-myc, σ1R-Nluc, σ1R KO cells). Anti-GAPDH bands are shown in green for equal loading. **(C,D)** Quantification of band intensities normalized to total signals within each lane for σ1R **(C)** and myc **(D)** blots. The intensities of bands up to hexamer were normalized to the total summation of the densities within the same lane. Due to a close migration pattern in a 10% polyacrylamide gel, higher order bands past hexamer were not resolvable for quantifications. **(E)** Radioligand binding properties of [^3^H] (+)-pentazocine for σ1R, σ1R-myc, and σ1R-Nluc (averaged curves are shown in Supplementary Figure S4). K_d_ and E_max_ values are not statistically different (one-way ANOVA). Each blot image is representative of n > 3 experiments that are averaged in bar graphs.

In WB assays, samples would typically be heated, often boiled, in a reducing buffer condition to allow efficient epitope interaction with an antibody in the following step. Indeed, the samples in previous WB assays of σ1R have also been subjected to heating (Gromek et al., 2014; Tsai et al., 2015; Sambo et al., 2017), unless using the native gel approach (Yano et al., 2018). In this study, we found that by keeping samples on ice before running them on sodium dodecyl sulfate–polyacrylamide gel electrophoresis (SDS-PAGE), we could observe strong-enough signals for homomeric σ1R interactions when using >5 μg plasmid DNA per 10-cm plate in transfection (Supplementary Figure S1D). Compared to samples receiving boiling treatment (100°C) that resulted in a single monomer band (∼25 kDa), the samples kept on ice showed a unique ladder-like pattern that corresponds to distinct bands from a monomer up to an octamer (∼200 kDa) of σ1R in WB (Supplementary Figure S2A). As expected, none of the bands were observed in the untransfected Δσ1R cells (Figure 1A). Therefore, we used the on-ice condition (4°C) for the rest of this study unless otherwise noted.

Using the same protocol, the extent of σ1R homomeric interactions at the endogenous expression level was tested in brain cortex preparation from mice. Notably, tetramer band has the highest intensity while dimer and very weak monomer densities are observed (Supplementary Figure S2C). Therefore, despite the differences in higher order bands compared to the HEK293T heterologous expression, the oligomerization of endogenously expressed σ1R can be detected by this protocol.

In addition to the B-5 (Santa Cruz) σ1R antibody, we chose three other commercially available σ1R antibodies based on their disparate target epitopes (Supplementary Table S1 and Supplementary Figures S1A–C). We compared the capabilities of these antibodies to detect distinct σ1R oligomer bands in our assay conditions. Similar to B-5, D4J2E (Cell Signaling; Supplementary Figure S1C) antibodies also showed a unique ladder-like pattern but not as distinct as B-5.

### The C-Terminally Tagged Constructs Show Distinct Oligomer Bands as Well

Previous reports suggest that C-terminal tagging of σ1R does not interfere with localization and function of σ1R (Hayashi and Su, 2003, 2007; Sambo et al., 2017). In addition to our previous σ1R construct used in the BRET assay in which Nluc is attached to the C-terminus (σ1R-Nluc) (Yano et al., 2018), to test the C-terminal tagging’s effect on the σ1R-σ1R interaction, we also generated the σ1R-myc construct, which has a shorter tag at the C-terminus. Both σ1R-Nluc and σ1R-myc exhibit ladder-like patterns similar to the unmodified WT σ1R; the bands range from ∼26 kDa (monomer) to ∼208 kDa (octamer) for σ1R-myc and ∼44 kDa (monomer) to ∼352 kDa (octamer) for σ1R-Nluc (Figure 1A). To confirm the same mobility and density of the bands visualized by an antibody targeting a different epitope, the anti-myc antibody was used against σ1R-myc as well. In this WB, the bands are generally brighter, which may indicate a stronger epitope affinity of the anti-myc antibody than that of the anti-σ1R antibody (Figure 1B).

To quantify the distribution of σ1R in each oligomeric population for each condition, we measured the intensity of each band (see section “Materials and Methods”). Similar patterns were observed across the unmodified and C-terminally tagged constructs (Figures 1C,D). The higher-order bands (i.e., tetramer, pentamer, and hexamer), which are larger than the trimer revealed by the σ1R crystal structures (Schmidt et al., 2016), are more intense for σ1R-Nluc and σ1R-myc than the unmodified construct (Figure 1C).

To confirm that the integrity of the ligand binding site and likely the C-terminal domain of the σ1R constructs are not affected by tagging or the WB condition, radioligand binding assay was conducted with [^3^H](+)-pentazocine. Under the same conditions as the WB (i.e., transfection and protein input), the unmodified and C-terminally-tagged constructs have virtually the same expression levels and similar K_d_ (Figure 1E and Supplementary Figure S4).

### The Unmodified WT and C-Terminally Tagged Constructs Show Similar Extents of Pharmacological Changes in Higher-Order Bands

**FIGURE 2:**
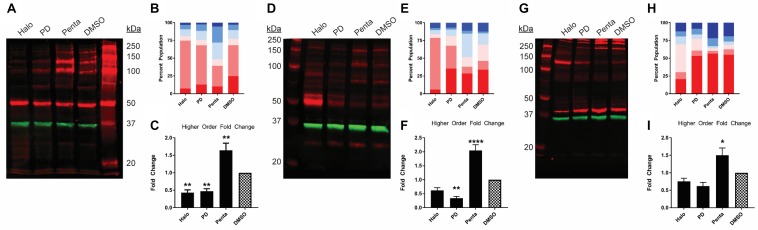
Pharmacological effects on SDS-PAGE mobility among the unmodified and C-terminally tagged σ1R constructs. Results for the unmodified **(A–C)**, C-terminally myc-tagged **(D–F)**, and C-terminally Nluc tagged **(G–I)** constructs are shown. **(A**,**D**,**G)** Red bands are visualized by anti-σ1R **(A,G)** or anti-myc **(D)** antibodies and green bands are visualized by anti-GAPDH **(A,D,G)** antibodies on 10% SDS-PAGE (protein standard, haloperidol, PD 144418, (+)-pentazocine, DMSO vehicle). **(B**,**E**,**H)** Quantification of band intensities normalized to total signals within each lane for σ1R **(B,H)** and myc **(E)** blots. **(C**,**F**,**I)** Pharmacological changes in higher order densities corresponding to tetramers, pentamers, and hexamers normalized to DMSO vehicle (haloperidol, PD 144418, (+)-pentazocine, DMSO vehicle). Each blot image is representative of n > 3 experiments that are averaged in bar graphs. One-way ANOVA followed by post hoc Tukey analysis (^∗^*p* < 0.05, ^∗∗^*p* < 0.01, and ^****^p < 0.001).

Previously, we have found that σ1R undergoes ligand-induced changes of the oligomerization states (Yano et al., 2018). Here, we investigated the effect of ligand binding on the migration pattern in WB. σ1R ligands were added to σ1R-expressing cells and incubated for 1 h before proceeding to sample preparation. 1 h incubation time was chosen to be consistent with the radioligand binding and BRET studies. For unmodified WT σ1R, both haloperidol and PD144418 decreased the intensities of the higher-order bands (i.e., tetramer, pentamer, and hexamer), while (+)-pentazocine increased their intensities (Figures 2A–C). The opposing effects of these ligands are consistent with our previous findings using BRET assays that categorized haloperidol, PD144418, and 4-PPBP into haloperidol-like ligands and (+)-pentazocine and PRE-084 into pentazocine-like ligands (Yano et al., 2018). Consistently, both σ1R-myc and σ1R-Nluc showed similar extents of changes in the higher-order bands, in which haloperidol and PD144418 decreased and (+)-pentazocine increased the relative densities of the higher-order bands (Figures 2D–I).

In contrast to samples prepared on ice, in the absence of any ligand, temperature increases in the sample buffer decreased the densities of the dimer and above bands, indicating that σ1R-σ1R interaction is disrupted by the high temperature (Supplementary Figure S2A), which was also observed when the experiments were carried out with mouse cortex preparation (Supplementary Figure S2C). In the presence of different ligands, the 70°C treatment completely monomerized σ1R as well, while the monomer densities across different ligand treatments are similar (Supplementary Figure S2B). Thus, the σ1R expression level is unlikely changed by the ligands, because changes in the total copy number would affect monomer band density.

### The N-Terminally Tagged myc-σ1R and Nluc-σ1R Exhibit Very Weak Higher Order Bands

**FIGURE 3:**
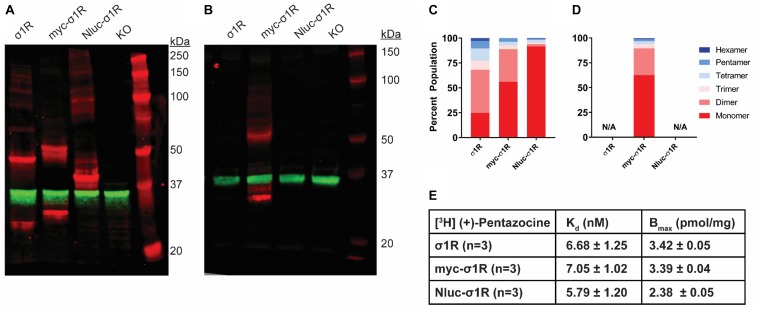
SDS-PAGE mobility comparison among the unmodified and N-terminally tagged σ1R constructs. **(A,B)** Red bands are visualized by anti-σ1R **(A)** or anti-myc **(B)** antibodies and green bands are visualized by anti-GAPDH **(A,B)** antibodies on 10% SDS-PAGE (protein standard, σ1R, myc-σ1R, Nluc-σ1R, σ1R KO cells). **(C,D)** Quantification of band intensities normalized to total signals within each lane for σ1R **(C)** and myc **(D)** blots. **(E)** Radioligand binding properties of [^3^H](+)-pentazocine for σ1R, myc-σ1R, and Nluc-σ1R (averaged curves are shown in Supplementary Figure S4). K_d_ and E_max_ values are not statistically different (one-way ANOVA). Each blot image is representative of n > 3 experiments that are averaged in bar graphs.

As opposed to the C-terminal tagging, it has been reported that N-terminal tagging of σ1R interferes with the localization of σ1R (Hayashi and Su, 2003). To study the effect of N-terminal tagging on the gel mobility of σ1R, in addition to our previously studied Nluc-σ1R (Yano et al., 2018), we generated the myc-σ1R construct. In our WB assay, using anti-σ1R antibody, the higher-order bands for both myc-σ1R and Nluc-σ1R have much less relative distributions than the unmodified construct (Figures 3A,C). When incubated with anti-myc antibody, the fraction of total density that was present in higher-order bands for myc-σ1R did not notably improve (Figures 3B,D). For Nluc-σ1R the reduced intensities in higher-order bands, are more apparent when compared to the density distribution of the C-terminally tagged σ1R-Nluc (Figures 1A,C, 3A,C). Even though the oligomerization states are drastically different from their corresponding C-terminal tagged constructs, radioligand binding results showed K_d_ and B_max_ are not significantly different from those of the unmodified construct (Figure 3E and Supplementary Figure S4).

### The N-Terminally Tagged Constructs Show Substantially Different Pharmacology From Unmodified WT σ1R

**FIGURE 4:**
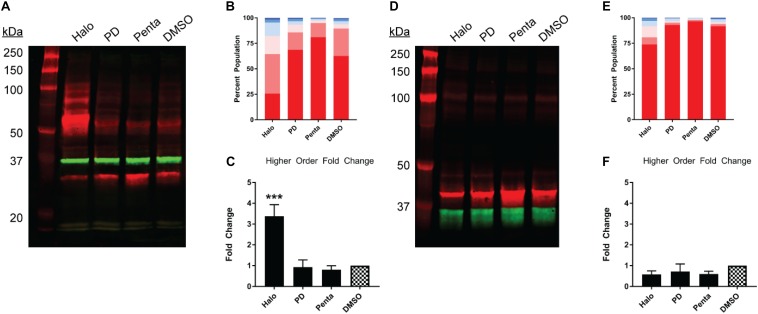
Pharmacological effects on SDS-PAGE mobility of the N-terminally tagged σ1R constructs. Results for the N-terminally myc-tagged **(A–C)**, and N-terminally Nluc-tagged **(D–F)** constructs are shown. **(A**,**D)** Red bands are visualized by anti-myc **(A)** or anti-σ1R **(D)** and green bands are visualized by anti-GAPDH **(A,D)** antibodies on 10% SDS-PAGE (protein standard, haloperidol, PD 144418, (+)-pentazocine, DMSO vehicle). **(B**,**E)** Quantification of band intensities normalized to total signals within each lane for myc **(B)** and σ1R **(E)** blots. **(C**,**F)** Pharmacological changes in higher order densities corresponding to tetramers and above bands normalized to DMSO vehicle (haloperidol, PD 144418, (+)-pentazocine, DMSO vehicle). Each blot image is representative of n > 3 experiments that are averaged in bar graphs. One-way ANOVA followed by post hoc Tukey analysis (^∗∗∗^*p* < 0.005).

Next, we studied the pharmacology of myc-σ1R and Nluc-σ1R in gel mobility to assess the effects by short and long N-terminal tagging. For myc-σ1R, haloperidol increased the higher-order bands while it diminished the monomer band (Figures 4A–C). PD144418 did not affect the relative density of each band, compared to the DMSO vehicle treated sample. (+)-pentazocine decreased the intensities of higher-order bands while increasing that of the monomer band. For Nluc-σ1R, haloperidol increased the higher order bands slightly while PD144418 showed effectively no change. (+)-pentazocine showed an increased monomer band (Figures 4D–F). Overall, the pharmacology of myc-σ1R and Nluc-σ1R are similar, i.e., haloperidol increased the densities of higher-order bands, while (+)-pentazocine increased that of monomer bands. However, these pharmacological trends are opposite to that of the unmodified σ1R (Figures 2A–C), indicating that the N-terminal tagging may interfere with how ligand binding propagates the conformational changes within σ1R or directly interferes with the formation of oligomers.

## Discussion

Sigma 1 receptor has been extensively studied at molecular level using biochemical and pharmacological approaches (Hayashi and Su, 2007; Narayanan et al., 2011; Gromek et al., 2014; Katz et al., 2016; Romero et al., 2016; Nguyen et al., 2017; Mavylutov et al., 2018). In this study, we investigated the effects of terminal tagging on σ1R oligomerization and pharmacology, and revealed the ability of N-terminal modification to interfere with the oligomerization of σ1R.

Sodium dodecyl sulfate–polyacrylamide gel electrophoresis WB is one of the most commonly used biochemical assays to quantify protein density. Because of detergent solubilization, protein-protein interactions in their native cellular environment can be disrupted in many instances. Here, however, by maintaining samples on ice, we were able to preserve homomeric σ1R-σ1R interactions and visualize discrete bands corresponding to oligomers as large as octamers by using an antibody (B-5) targeting an epitope at residues 136-169 of σ1R. As a technical note, after the antibody has been stored for a long time, the antibody staining becomes dimmer and the higher-order bands cannot be visualized, potentially due to degradation (Supplementary Figures S1E,F). Interestingly, the other antibodies against distinct epitopes from different vendors resulted in different visualization patterns indicating the corresponding epitopes may be differentially exposed in different oligomerization states, while residue 136-169 might always be exposed. Although intriguing, these antibodies were not further pursued because of their significantly lower band intensities, which could be due to insufficient epitope affinities. In general, the WB method described herein is deemed useful to study σ1R pharmacology since ligand-induced conformational changes, particularly in higher-order bands, can be quantitatively evaluated. Nevertheless, we could not exclude the possibility that σ1R-interacting proteins may co-migrate with σ1R and result in some of the observed bands, though such contribution is likely minimal given the significantly higher heterologous expression level of σ1R compared to the other endogenous proteins in this study.

We have previously reported a BRET-based proximity assay to study σ1R pharmacology in HEK293T cells (Yano et al., 2018). The C-terminally tagged σ1R-Nluc construct used in that study showed a similar response to ligands in the migration pattern of higher-order bands as the unmodified σ1R, demonstrating that BRET responses very likely reflect the configurational changes in σ1R oligomerization observed in the unmodified σ1R. In current study, we used Δσ1R HEK293T cells. By repeating the BRET experiment in Δσ1R HEK293T cells transfected with σ1R-Nluc and σ1R-Venus (Supplementary Figure S3), we found the σ1R pharmacology effectively remained the same as the previously reported BRET results in HEK293T cells (Yano et al., 2018). Given that in the BRET assays, haloperidol and PD144418 increased BRET signals while in the WB assays, they decreased higher-order band densities and increased lower-order band densities, we speculate that the BRET readouts recapitulate the changes in the lower-order populations. In that regard, the dimer and trimer populations comprise 48.9% in haloperidol and 24.3% in PD144418, compared to 13.7% in DMSO (Figure 2H). The pattern can be correlated with the BRET_max_ order, in which haloperidol shows higher BRET_max_ than PD144418 (Supplementary Figure S3).

We observed that the total signal (i.e., summation of band densities up to hexamer) increased with (+)-pentazocine (38%) and decreased with haloperidol (−25%) or PD144418 (−23%) compared to DMSO lane (Figure 2A). Since there is no change in σ1R expression level across different ligand conditions (Supplementary Figure S2B), these changes in total signal, primarily due to changes in the higher-order bands, are caused by changes in either the copy number and/or changes in epitope accessibility in higher-order oligomers. If there is an increase in copy number of σ1R in higher order bands, the increased populations should equal the collective decrease in lower-order bands. However, as we only observed a weak complementary decrease in lower-order bands, in addition to increased copy number, we postulate that changes in the epitope affinity in higher-order populations of σ1R plays a role in increased higher-order band intensities as well.

In addition, we found that the N-terminal modification limits higher-order interactions of σ1R in our solubilization condition, consistent with the previously reported detrimental effects of N-terminal tagging or point mutation on the cellular localization and function (Hayashi and Su, 2003). It is noteworthy that there is a putative Arg-Arg ER retention signal (Michelsen et al., 2005) at the N-terminus of σ1R. We propose that the steric hindrance of the N-terminally tagged protein may interfere with the recognition of Arg-Arg tandem residues at the seventh and eighth positions. This interference is potentially akin to some other membrane proteins that escape from ER retention due to its Arg-Arg motif masked by nearby domains [e.g., GABA_*B*_ receptor (Frangaj and Fan, 2018)]. Despite its probable mislocalization, the folding of σ1R is likely only be modestly affected by the N-terminal tagging, considering the similar binding K_d_ compared to that of the unmodified construct. Therefore, the reduced σ1R-σ1R interaction may arise from the membrane environment in which σ1R is localized such that the σ1R density may be too low to find an interacting partner. Further, while the exterior of the barrel motif of the C-terminal domain has been revealed by the crystal structures to be the interface for trimer, it is likely other part of the of the protein involves in forming other oligomer states, such as the N-terminus and N-terminal transmembrane helix^1^, which may be disrupted by the N-terminal tagging.

## Data Availability Statement

All datasets generated for this study are included in the article/Supplementary Material.

## Ethics Statement

The animal study was reviewed and approved by the Institutional Care and Use Committee of the Intramural Research Program, National Institute on Drug Abuse.

## Author Contributions

HY and LS designed the study. LL, SN, and HY performed the experiments. All authors took part in interpreting the results. HY wrote the initial draft, with LL and LS participating in revising the manuscript.

## Conflict of Interest

The authors declare that the research was conducted in the absence of any commercial or financial relationships that could be construed as a potential conflict of interest.

## Footnotes

1 Abramyan, A. M., Yano, H., Xu, M., Liu, L., Naing, S., Fant, A. D., et al. (Submitted). *The Mutation of Glu102 Disrupts Higher-Order Oligomerization of the Sigma 1 Receptor*.

## References

[B1] Frangaj A. Fan Q. R. (2018). Structural biology of GABAB receptor. *Neuropharmacology* 136 68–79. 10.1016/j.neuropharm.2017.10.011 29031577 PMC5897222

[B2] Gromek K. A. Suchy F. P. Meddaugh H. R. Wrobel R. L. LaPointe L. M. Chu U. B. (2014). The oligomeric states of the purified sigma-1 receptor are stabilized by ligands. *J. Biol. Chem.* 289 20333–20344. 10.1074/jbc.M113.537993 24847081 PMC4106346

[B3] Hayashi T. Su T. P. (2003). Sigma-1 receptors (sigma(1) binding sites) form raft-like microdomains and target lipid droplets on the endoplasmic reticulum: roles in endoplasmic reticulum lipid compartmentalization and export. *J. Pharmacol. Exp. Ther.* 306 718–725. 10.1124/jpet.103.051284 12730355

[B4] Hayashi T. Su T. P. (2007). Sigma-1 receptor chaperones at the ER-mitochondrion interface regulate Ca(2+) signaling and cell survival. *Cell* 131 596–610. 10.1016/j.cell.2007.08.036 17981125

[B5] Katz J. L. Hiranita T. Hong W. C. Job M. O. McCurdy C. R. (2017). A role for sigma receptors in stimulant self-administration and addiction. *Handb. Exp. Pharmacol.* 244 177–218. 10.1007/164_2016_94 28110353

[B6] Katz J. L. Hong W. C. Hiranita T. Su T. P. (2016). A role for sigma receptors in stimulant self-administration and addiction. *Behav. Pharmacol.* 27 100–115. 10.1097/FBP.0000000000000209 26650253 PMC4779716

[B7] Maurice T. Su T. P. (2009). The pharmacology of sigma-1 receptors. *Pharmacol. Ther.* 124 195–206. 10.1016/j.pharmthera.2009.07.001 19619582 PMC2785038

[B8] Mavylutov T. Chen X. Guo L. Yang J. (2018). APEX2- tagging of Sigma 1-receptor indicates subcellular protein topology with cytosolic N-terminus and ER luminal C-terminus. *Protein Cell* 9 733–737. 10.1007/s13238-017-0468-5 28929457 PMC6053353

[B9] Michelsen K. Yuan H. Schwappach B. (2005). Hide and run. Arginine-based endoplasmic-reticulum-sorting motifs in the assembly of heteromultimeric membrane proteins. *EMBO Rep.* 6 717–722. 16065065 10.1038/sj.embor.7400480 PMC1369147

[B10] Narayanan S. Bhat R. Mesangeau C. Poupaert J. H. McCurdy C. R. (2011). Early development of sigma-receptor ligands. *Future Med. Chem.* 3 79–94. 10.4155/fmc.10.279 21428827

[B11] Nguyen L. Lucke-Wold B. P. Mookerjee S. Kaushal N. Matsumoto R. R. (2017). Sigma-1 receptors and neurodegenerative diseases: towards a hypothesis of sigma-1 receptors as amplifiers of neurodegeneration and neuroprotection. *Adv. Exp. Med. Biol.* 964 133–152. 10.1007/978-3-319-50174-1_10 28315269 PMC5500918

[B12] Romero L. Merlos M. Vela J. M. (2016). Antinociception by sigma-1 receptor antagonists: central and peripheral effects. *Adv. Pharmacol.* 75 179–215. 10.1016/bs.apha.2015.11.003 26920013

[B13] Sambo D. O. Lin M. Owens A. Lebowitz J. J. Richardson B. Jagnarine D. A. (2017). The sigma-1 receptor modulates methamphetamine dysregulation of dopamine neurotransmission. *Nat. Commun.* 8:2228. 10.1038/s41467-017-02087-x 29263318 PMC5738444

[B14] Schmidt H. R. Betz R. M. Dror R. O. Kruse A. C. (2018). Structural basis for sigma1 receptor ligand recognition. *Nat. Struct. Mol. Biol.* 25 981–987. 10.1038/s41594-018-0137-2 30291362 PMC6261271

[B15] Schmidt H. R. Zheng S. Gurpinar E. Koehl A. Manglik A. Kruse A. C. (2016). Crystal structure of the human sigma1 receptor. *Nature* 532 527–530. 10.1038/nature17391 27042935 PMC5550834

[B16] Su T. P. Su T. C. Nakamura Y. Tsai S. Y. (2016). The sigma-1 receptor as a pluripotent modulator in living systems. *Trends Pharmacol. Sci.* 37 262–278. 10.1016/j.tips.2016.01.003 26869505 PMC4811735

[B17] Tsai S. Y. Chuang J. Y. Tsai M. S. Wang X. F. Xi Z. X. Hung J. J. (2015). Sigma-1 receptor mediates cocaine-induced transcriptional regulation by recruiting chromatin-remodeling factors at the nuclear envelope. *Proc. Natl. Acad. Sci. U.S.A.* 112 E6562–E6570. 10.1073/pnas.1518894112 26554014 PMC4664336

[B18] Urizar E. Yano H. Kolster R. Gales C. Lambert N. Javitch J. A. (2011). CODA-RET reveals functional selectivity as a result of GPCR heteromerization. *Nat. Chem. Biol.* 7 624–630. 10.1038/nchembio.623 21785426 PMC3158273

[B19] Weissman A. D. Su T. P. Hedreen J. C. London E. D. (1988). Sigma receptors in post-mortem human brains. *J. Pharmacol. Exp. Ther.* 247 29–33. 2845055

[B20] Yano H. Bonifazi A. Xu M. Guthrie D. A. Schneck S. N. Abramyan A. M. (2018). Pharmacological profiling of sigma 1 receptor ligands by novel receptor homomer assays. *Neuropharmacology* 133 264–275. 10.1016/j.neuropharm.2018.01.042 29407216 PMC5858991

