# Supplementary material for: The Effects of Terminal Tagging on Homomeric Interactions of the Sigma 1 Receptor
Source: Front Neurosci. 2019 Dec 19;13:1356. doi: 10.3389/fnins.2019.01356 (PMC6930887; doi:10.3389/fnins.2019.01356)

Supplementary Figure 1. Immuno-reactivities of commercially available σ1R antibodies. **A-C**. Green and red bands are visualized by anti-σ1R (**A**. AB 53852, Abcam, **B**. AB 223702, Abcam, **C**. DHJ2E, Cell Signaling) and anti-actin antibodies respectively on 10% SDS-PAGE. **D**. Endogenous σ1R expressed in WT HEK293T cells or increasing amounts of σ1R expressed in σ1R KO cells (1, 5, 8 μg) are detected by the anti-σ1R antibody (B-5, Santa Cruz) in red along with GAPDH loading control in green. **E-F**. Immuno-reactivities are diminished over a prolonged storage time (**E**. ~1 month vs. **F**. > 6 months in 4 °C). Each blot image is representative of n > 3 experiments.

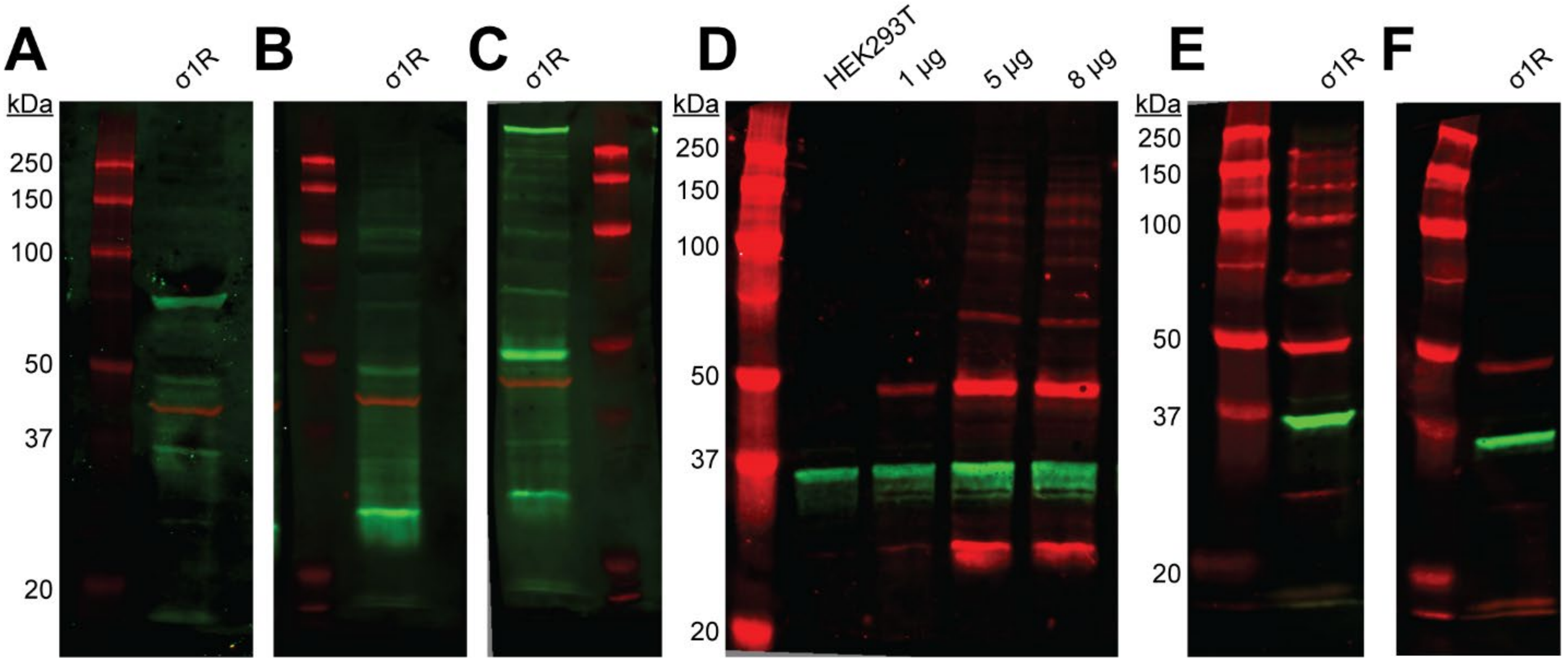

Supplementary Table 1.

Product information and dilutions of primary and secondary antibodies

	Product ID, Vendor	Epitope (residue numbers)	Dilution
Primary antibody			
σ1R (mouse monoclonal)	B-5, Santa Cruz	136-169	1:1,000
σ1R (rabbit polyclonal)	AB 53852, Abcam	C-terminus	1:625
σ1R (rabbit polyclonal)	AB 223702, Abcam	35-88	1:250
σ1R (rabbit monoclonal)	DHJ2E, Cell Signaling	around 137	1:1,000
GAPDH (rabbit monoclonal)	14C10, Cell Signaling	C-terminus	1:2,000
Actin (mouse monoclonal)	C-2, Santa Cruz	357-375	1:2,000
Myc (mouse monoclonal)	9B11, Cell Signaling	410-419	1:1,000
Secondary antibody			
Donkey α mouse	IRDye 680RD, LI-COR		1:5,000; 1:10,000
Goat α rabbit	IRDye 800CW, LI-COR		1:10,000; 1:5,000

Supplementary Figure 2. Protein sample preparation at different temperatures. **A**-**B**. Sample preparation from HEK 293T cells, red and green bands are visualized by anti-σ1R and anti-GAPDH antibodies respectively on 10% SDS-PAGE. **A**. Samples were prepared in increasing temperatures (4, 40, 70, and 100 °C). **B**. Ligand-treated samples were heated at 70 °C for 15 min (protein standard, haloperidol, PD 144418, (+)-pentazocine, DMSO vehicle). **C**. Samples from mouse brain cortex were prepared in 4 and 70°C, and were visualized by anti-σ1R antibody. Each blot image is representative of n > 3 experiments.

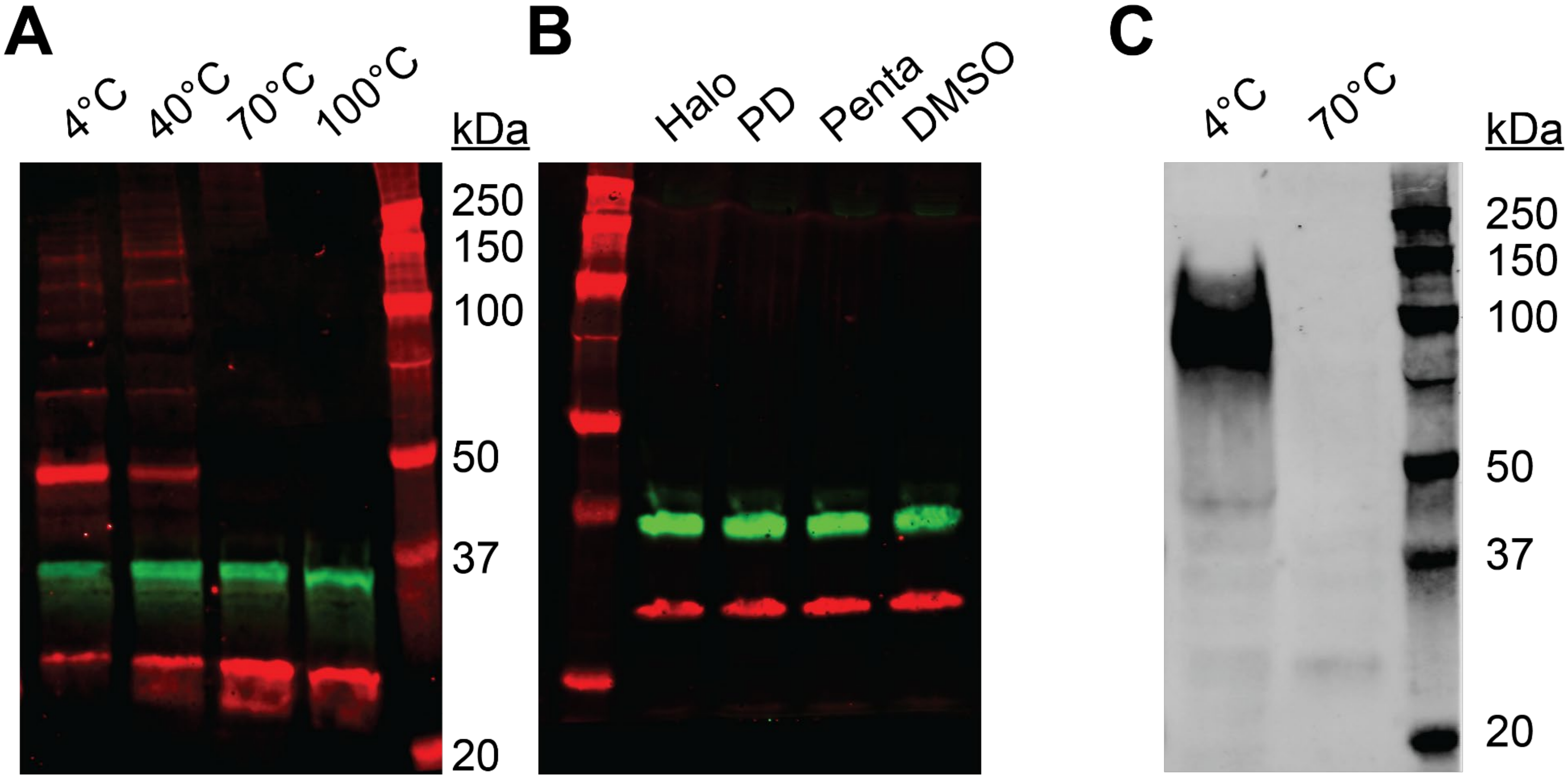

Supplementary Figure 3. Drug induced changes of σ1R homomer BRET. Drug induced BRET between the C-terminally tagged σ1R-Nluc and σ1R-Venus in σ1R KO cells is detected at 60 min for (+)-pentazocine (red), haloperidol (blue), and PD144418 (cyan). Data represents mean±S.E.M. (n=5 or more).

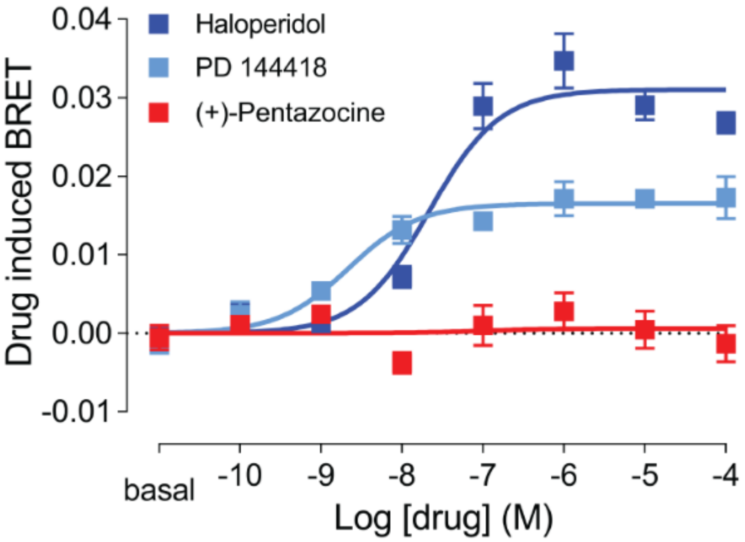

Supplementary Figure 4. Homologous radioligand competition binding of [^{3}H](+)-pentazocine for σ1R (black), σ1R-myc (orange), σ1R-Nluc (red), myc-σ1R (blue), and Nluc-σ1R (purple). Each curve is the average of n = 3 using one-site fitting.

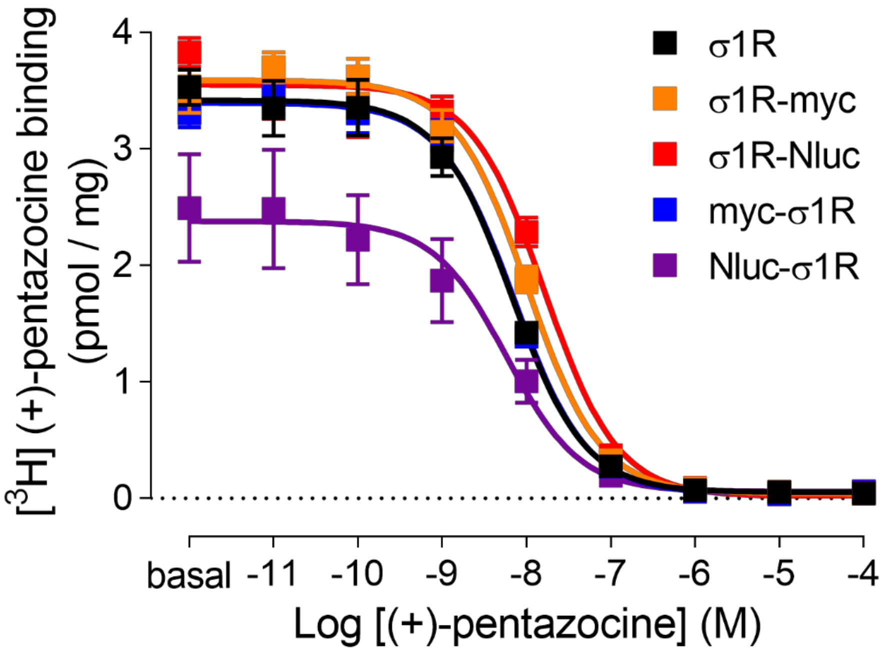

Supplement: Supplementary file 1 [file Data_Sheet_1.pdf]
